# Sol-Gel Dipping Devices for H_2_S Visualization

**DOI:** 10.3390/s23042023

**Published:** 2023-02-10

**Authors:** Maria Strianese, Giovanni Ferrara, Viktoriia Vykhovanets, Naym Blal, Daniela Guarnieri, Alessandro Landi, Marina Lamberti, Andrea Peluso, Claudio Pellecchia

**Affiliations:** Dipartimento di Chimica e Biologia “Adolfo Zambelli”, Università degli Studi di Salerno, Via Giovanni Paolo II, 132, 84084 Fisciano, Italy

**Keywords:** fluorescence, hydrogen sulfide, portable sensors

## Abstract

In this contribution we report the synthesis and full characterization, via a combination of different spectroscopies (e.g., ^1^H NMR, UV-vis, fluorescence, MALDI), of a new family of fluorescent zinc complexes with extended π-conjugated systems, with the final aim of setting up higher performance H_2_S sensing devices. Immobilization of the systems into a polymeric matrix for use in a solid-state portable device was also explored. The results provided proof-of-principle that the title complexes could be successfully implemented in a fast, simple and cost-effective H_2_S sensing device.

## 1. Introduction

The discovery that in addition to its traditional toxic role, hydrogen sulfide (H_2_S) [1] is an endogenously produced molecule which is involved in a variety of key biological processes within the human body [2,3], induced several research groups to try to address hydrogen sulfide’s (bio) reactivity in more detail. The complex biological chemistry of H_2_S involves numerous cellular targets in both physiological and pathological conditions and the transition metals constitute logical targets of reactivity given their affinity for the Lewis-basic sulfur atom of hydrogen sulfide. The role of H_2_S as a gaseous signaling molecule, together with nitric oxide (NO) and carbon monoxide (CO), is by now commonly acknowledged [4,5,6] and akin to NO and CO, H_2_S and its conjugate base, the hydrosulfide ion (HS^−^), are willing to coordinate to transition metals, however, the coordination chemistry of H_2_S/HS^−^ has been much less studied. The relative scarcity of studies concerning the metal complexes of H_2_S/HS^−^ is mainly due to the difficulties associated with the reactivity of the molecule, including its redox reactivity, and the different chemistry associated with the specific protonation state. Notwithstanding this, the study of the bioinorganic chemistry of hydrogen sulfide has been ongoing for some time [7,8,9,10,11,12,13,14,15] and still represents an active area of study [16]. Studies demonstrating the signaling pathways involving the metalloproteins together with the evidence implicating H_2_S/HS^−^ in an increasing number of biological processes inspired most of the recent studies on the metal-based chemistry of H_2_S. Both natural and synthetic systems have been proposed as new examples of metal complexes capable of coordinating H_2_S/HS^−^ [17,18,19,20,21,22,23,24,25,26,27,28,29,30,31,32]. Considering the very flexible and attractive optical properties of salen-based zinc complexes [33,34,35,36,37,38], and motivated by our interest in the coordination of H_2_S to the transition metals, we focused on salen-based zinc systems and studied their reactivity with H_2_S/HS^−^ [39,40,41,42,43]. Our studies provide evidence that the zinc-salen systems constitute a suitable platform of systems that are able to stabilize hydrosulfide coordination at the zinc center and can act as very efficient HS^−^ sensing constructs via a ‘coordinative-based’ mechanism [44].

**Scheme 1:**
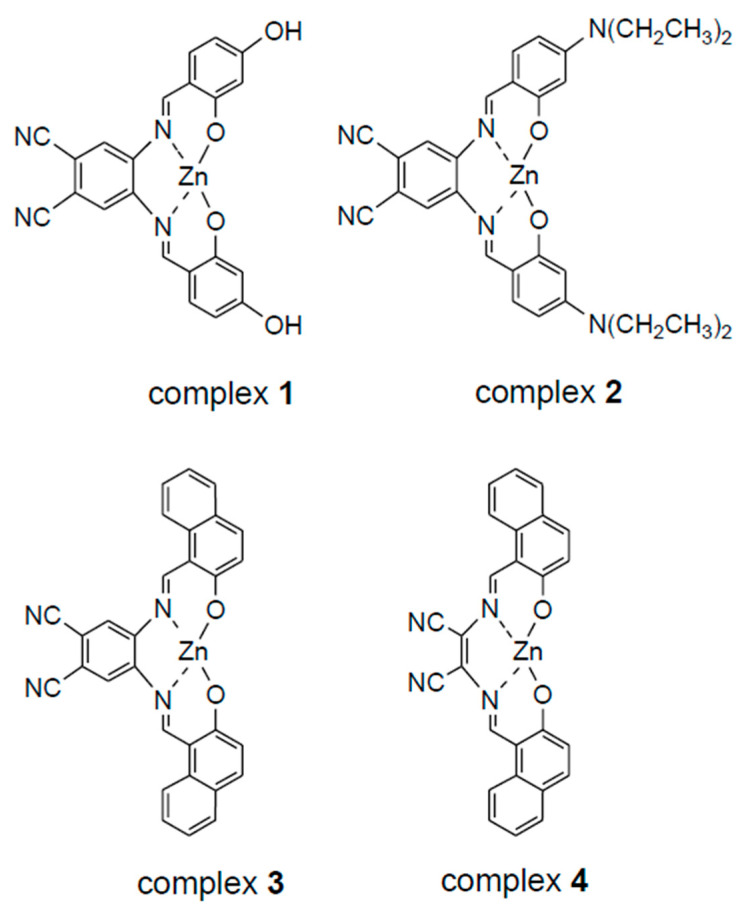
Structures of the complexes studied in this work.

In the present study, we explored whether enhancing the size of the π-conjugated system of the bridge between the zinc-chelating nitrogens and/or of the phenolate moieties would affect the zinc hydrosulfide stabilization and additionally if the different ligand structures would improve the fluorescence and colorimetric properties of the related complexes as HS^−^ sensors. We also wanted to investigate whether increasing the rigidity of the bridge between the two nitrogen donor atoms, by introducing a more conjugated spacer (e.g., passing from a diaminomalonitrile (DAMN) [41] to a phthalonitrile bridge), would tune the fluorescence responses in the presence of HS^−^ and the overall photophysical properties of the resulting complexes. To address this aim, we synthesized a new family of salen-based zinc complexes, as shown in Scheme 1.

In our long-term research concerning the implementation of higher performance sensors for H_2_S determination, we explored the possibility of immobilizing the title complexes into silica matrices aimed at setting-up more stable and portable sensors.

## 2. Experimental Section

### 2.1. Materials

Chemicals used for the synthetic work were obtained from Sigma-Aldrich or Strem Chemicals and were of reagent grade. They were used without further purification. NaSH (Alfa Aesar) in aqueous solution was used as HS^−^ source to the end concentration specified in the figure captions. Syntheses of complexes **1**–**4** were achieved by following literature procedures [37,38].

### 2.2. General

HR MALDI and ESI mass spectra were registered by using a Bruker solariX XR Fourier transform ion cyclotron resonance (FT-ICR) mass spectrometer (Bruker Daltonik GmbH, Bremen, Germany) equipped with a 7 T refrigerated actively-shielded superconducting magnet (Bruker Biospin, Wissembourg, France). The samples were ionized in positive ion mode using either the MALDI ion or the ESI ion source. The mass range was set to *m*/*z* 150–2000. The laser power was 15% and 15 laser shots were used for each scan. Mass spectra were calibrated externally using a mix of peptide clusters in MALDI ionization positive (or negative) ion mode. A linear calibration was applied.

NMR spectra were registered on a Bruker AVANCE 400 NMR instrument (^1^H NMR, 400.13 MHz; ^13^C NMR, 100.62 MHz) or on a 600 MHz spectrometer [600 (^1^H NMR) and 150 MHz (^13^C NMR)] using 5 mm o.d. NMR tubes. The chemical shifts were reported in δ (ppm) referenced to SiMe_4_. Typically, 5 mg of the complex in 0.5 mL of the solvent were used for each experiment.

**Synthesis of complexes 1 and 2.** A mixture of the appropriate aldehyde (2 mmol) and 4,5-diaminophthtalonitrile (0.158 g, 1 mmol) in 50 mL of absolute ethanol was left under stirring for 1 h at room temperature. Then 1 eq of Zn(CH_3_COO)_2_*2H_2_O (0.219 g, 1 mmol) in 150 mL of absolute ethanol was added and the reaction vessel was left under stirring and at reflux overnight.

**Synthesis of complexes 3 and 4.** A mixture of 2 eq of 2-hydroxy-1-naphthaldehyde (0.344 g, 2 mmol) and 1 eq of Zn(CH_3_COO)_2_*2H_2_O (0.219 g, 1 mmol) in *N,N*-dimethylformamide (DMF) was left under stirring for 30 min at room temperature. Then diamine (1 mmol) was added and the mixture was left under stirring overnight at room temperature.

**Characterization of complex 1**. A golden-sheet-like solid was recovered by filtration on common filter paper and dried under vacuum (0.345 g, yield 75 %). ^1^H NMR [400 MHz, DMSO-d_6_]: δ = 10.11 (s, 2H, O***H***), 8.93 (s, 2H, ***H*** phthalonitrile), 8.48 (s, 2H, C***H***=N), 7.20 (d, J = 8.7 Hz, 4H, ***H*** aromatic), 6.11 (d, 2H, J = 8.6 Hz, ***H*** aromatic), 6.04 (s, 2H, ***H*** aromatic). MS (MALDI CH_3_CN with 1% DMSO): *m*/*z* (%) 461.02 (100) [complex **1** H]^+^.

**Characterization of complex 2.** A dark-orange precipitate was recovered by filtration washed with cold methanol and dried under vacuum (0.285 g, yield 50%). ^1^H NMR [300 MHz, DMSO-d_6_]: δ = 8.78 (s, 2H, ***H*** phthalonitrile), 8.36 (s, 2H, C***H***=N), 7.14 (d, 2H, J = 9.2 Hz, ***H*** aromatic), 6.19 (d, 2H, J = 9.2 Hz, ***H*** aromatic), 5.81 (s, 2H, ***H*** aromatic), 3.38 (m, 8H, N(C***H***_2_CH_3_)_2_; 1.15 (m, 12H, N(CH_2_C***H***_3_)_2_). MS (MALDI CH_3_OH): m/z (%) 471.17 (100) [complex **2** H]^+^.

**Characterization of complex 3**. An orange-brown solid was recovered by filtration on common filter paper, this was then washed with cold methanol and dried under vacuum (0.396g, yield 75 %). ^1^H NMR [300 MHz, DMSO-d6]: δ = 9.83 (s, 2H, C***H***=N), 8.95 (s, 2H, ***H*** phthalonitrile), 8.59 (d, 2H, J = 8.2 Hz, ***H*** naphthaldehyde), 7.87 (d, J = 8.7 Hz, 2H, ***H*** naphthaldehyde), 7.72 (d, 2H, J = 7.6 Hz, ***H*** naphthaldehyde), 7.52 (t, 2H, J = 8.1 Hz, ***H*** naphthaldehyde), 7.29 (t, 2H, J = 7.6 Hz, ***H*** naphthaldehyde), 6.99 (d, 2H, J = 9.3 Hz, ***H*** naphthaldehyde). MS (MALDI THF): m/z (%) 567.01 [complex **3** K]^+^; 551.04 [complex **3** Na]^+^; 529.06 [complex **3** H]^+^.

**Characterization of complex 4**. A purple solid was recovered by filtration on common filter paper, this was then washed with cold methanol and dried under vacuum (0.359 g, yield 75 %). ^1^H NMR [300 MHz, DMSO-d6]: δ = 9.30 (s, 2H, C***H***=N), 8.09 (d, 2H, J = 8.4 Hz, ***H*** naphthaldehyde), 7.93 (d, 2H, J = 9.4 Hz ***H*** naphthaldehyde),7.75 (d, 2H, J = 7.8 Hz, ***H*** naphthaldehyde), 7.56 (t, 2H, J = 7.6 Hz, ***H*** naphthaldehyde), 7.32 (t, 2H, J = 7.3 Hz, ***H*** naphthaldehyde), 7.00 (d, 2H, J = 9.4 Hz, ***H*** naphthaldehyde). MS (MALDI THF): m/z (%) 478.04 (100) [complex **4**]^+^; 501.03 [complex **4** Na]^+^.

**Complexes encapsulation in tetramethoxysilicate (TMOS) sol gel.** The preparation of silica gels and the encapsulation of complexes were undertaken with pure TMOS [45]. TMOS (15.22 g) was mixed with milliQ water (3.38 g) in a 1:2 molar ratio followed by the addition of 20 µL of 10 mM HCl. The reaction mixture was sonicated for 20 min. Then roughly 1 mL of complex solution in DMSO (end conc. of complex: 5–10 µM) was added to the mixture. Before gelation 150 µL of the sol solution was quickly poured onto a home-made device (8 × 30 mm^2^ quartz slide Heraeus 3 quality with a 1 mm thickness) yielding a roughly 0.6 mm thick sol–gel layer on top of the quartz slide. Activation of quartz slides with “Piranha solution” (30% H_2_O_2_ and concentrated H_2_SO_4_ in a 1:3 volume ratio) was performed before pouring the sol solution on top. [Caution: “Piranha solution” is highly corrosive and should be handled with extreme care]. After drying, the sol-gels were analyzed under an ultraviolet lamp (Spectroline ENF-240C/FE) working at 365 nm wavelength irradiation.

**Absorbance and fluorescence measurements.** A Cary-50 Spectrophotometer with a 1 cm quartz cuvette (Hellma Benelux bv, Rijswijk, The Netherlands) and a slit-width equivalent to a bandwidth of 5 nm was used for the absorption spectra. Fluorescence spectra were measured on a Cary Eclipse Spectrophotometer in a 10 × 10mm^2^ airtight quartz fluorescence cuvette (Hellma Benelux bv, Rijswijk, The Netherlands) with an emission band-pass of 10 nm and an excitation band-pass of 5 nm. Both absorption and fluorescence measurements were performed in DMSO solutions at 25 °C. Fluorescence emission spectra were registered by exciting the samples at a specific wavelength (as stated in the figure captions).

Fluorescence quantum yield (Φ_F_) values were determined in optically diluted solutions of the samples by using as standards the commercial dye Cy3 NHS (Φ_F_ = 0.15 in MilliQ water) for complex **1** and Cy5 NHS (Φ_F_ = 0.28 in MilliQ water) in the case of complex **4**, according to the equation [46]:

Φ_F_^s^ = Φ_F_^r^ (*I*_s_/*I*_r_)(*A*_r_/*A*_s_)(*η*_s_/*η*_r_)^2^ (1)

where indexes s and r denote the sample and reference, respectively. *I* stands for the integrated emission intensity, *A* is the absorbance at the excitation wavelength, and *η* is the refractive index of the solvent. The optical density of complexes **1** and **4** and standards was kept below 0.1. The uncertainty in the determination of Φ_F_ is ±15%.

**NMR characterization of the complexes 1–4 upon addition of HS^−^.** NMR samples were prepared by charging the tubes with the free complexes solutions in DMSO-*d_6_* then NaSH solid or in solution (to the end concentrations specified in the figure captions) was added and the spectra registered.

**Computational details**. All electronic computations were carried out at the density functional level of theory using the range separated hybrid functional CAM-B3LYP with TZVP basis set as implemented in the Gaussian package (G16) [47]. This combination of functional and basis set was chosen because it results in accurate predictions, as discussed in previous works [48]. Time dependent DFT (TD-DFT) was employed for treating all excited states. Spin-orbit coupling elements were computed by PySOC code [49,50]. Effects due to solvent polarization [51] were included by the polarizable continuum model (PCM) [52].

**Cell culture and cell-conditioned media preparation.** HepG2 cells (Human hepatocellular liver carcinoma cell line) were grown in Minimum Essential Medium (MEM) supplemented with 10% fetal bovine serum (FBS), 2 mM Glutamine, 1 mM non-essential amino acids, and 1% antibiotics (penicillin/streptomycin, 100 U/mL). Cells were maintained in a humidified incubator at 37 °C in 5% CO_2_/95% air. To prepare cell-conditioned HBSS media, approximately 5 × 10^5^ cells were seeded in each well of a 6-well plate and cultured with 1 mL of HBSS for 24 h. To induce endogenous production of HS^−^, cells were treated with 800 µM of H_2_O_2_ for 1 h. After the incubation, conditioned media were collected and used for testing sol-gel devices as described above.

## 3. Results and Discussion

Complexes **1**–**4** were easily prepared in a one-step procedure by reacting the appropriate salicylaldehyde and the corresponding diamine in a 2:1 ratio with one equivalent of zinc acetate dihydrate, by following the literature procedures [37,38,53]. The ^1^H NMR and high-resolution MALDI Fourier transform ion cyclotron resonance mass (HR MALDI-FT-ICR) experiments assessed the purity of the complexes under investigation (see Figures S1–S8). The protonic spectra exhibited sharp and narrow signals with a highly symmetric pattern, confirming the proposed structures (Figures S1–S4). In the MALDI spectra (Figures S5–S8) the major peaks at 461.021 m/z units for complex **1**, at 571.176 m/z units for complex **2,** at 529.063 *m*/*z* units for complex **3** and at 478.040 *m*/*z* units for complex **4** corresponded to the monomeric species: no peaks ascribable to the dimeric species were found.

**Figure 1:**
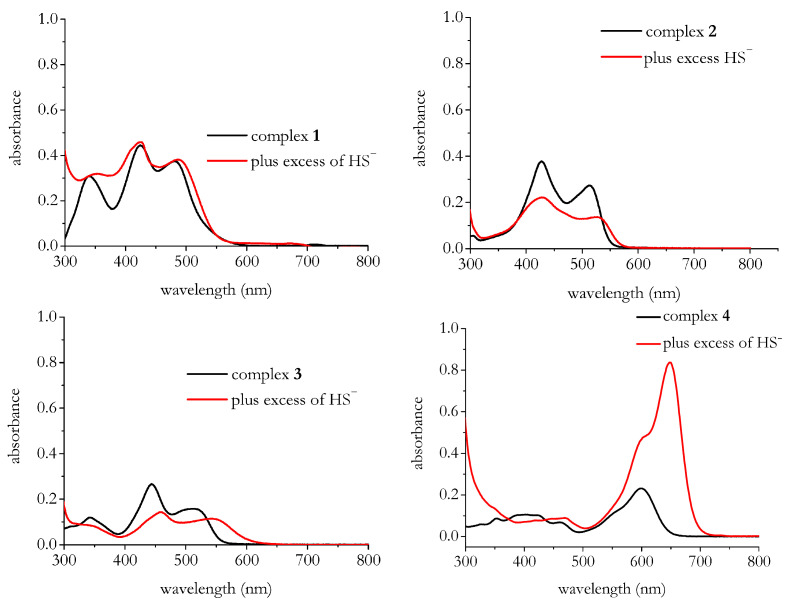
Electronic absorption spectra of complexes **1**–**4** with and without the addition of 50 µM of NaSH. Spectra were recorded in DMSO at room temperature. [complexes] = 10 µM.

After characterization of complexes **1**–**4** we commenced screening of the optical properties, to assess their potential use in the colorimetric and fluorometric recognition of HS^−^. Figure 1 displays the UV-vis spectra of the title complexes before and after the treatment with an excess of HS^−^.

As clearly visible from Figure 1, all the complexes under investigation absorbed in the blue-green section of the electromagnetic spectrum and exhibited a change of the initial UV-visible spectrum upon interaction with HS^−^ which suggests the formation of a new species.

In the case of complex **4** a significant naked-eye color variation was also observed (see Figure S9).

**Figure 2:**
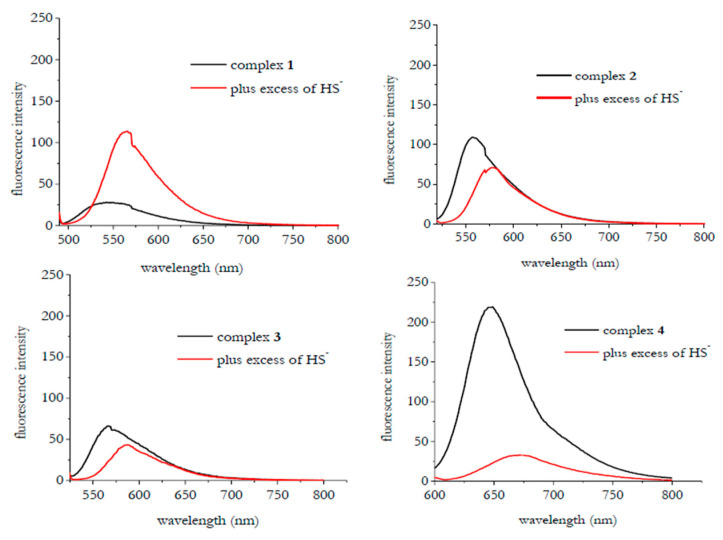
Emission spectra of complexes **1**−**4** before and after the addition of 5 equiv of NaSH. [Complexes **1**−**4**] = 1 × 10^−5^ M; [NaSH] = 5 × 10^−5^ M. All spectra were measured in DMSO with λ_exc_ = 481 nm for complex **1**; λ_exc_ = 513 nm for complex **2**; λ_exc_ = 516 nm for complex **3;** λ_exc_ = 598 nm for complex **4**.

Subsequently, we tested the fluorescence responses in the presence of HS^−^. The outcome of these experiments is shown in Figure 2. The most emissive complex of the series was complex **4** which was also the complex exhibiting a more red-shifted emission with respect to the others under investigation.

The addition of HS^−^ resulted in a significant fluorescence switching for all of the complexes under investigation. In particular, complex **1** experienced a turn-on of the initial fluorescence intensity whereas complexes **2–4** harnessed a fluorescent quenching.

To further test the sensing abilities of the complexes under investigation for H_2_S visualization/detection, we checked whether the binding of HS^−^ was reversible, as observed with probes which we and others had proposed in the past [22,25,28]: reversibility is a key property for application in real measurements.

**Figure 3:**
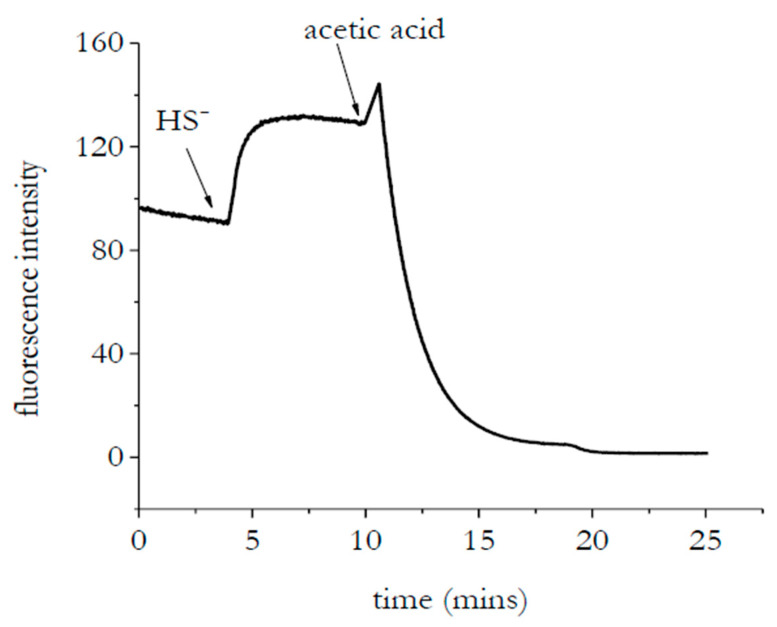
Emission spectrum of complex **1** (λ_ex_ = 481 nm; λ_em_ = 550 nm), upon addition of 5 equiv of NaSH and upon addition of 10 equiv of acetic acid. [Complex **1**] = 1 × 10^−5^ M; [NaSH] = 5 × 10^−5^ M. Spectrum was measured in DMSO.

More precisely, to assess the reversibility of HS^−^ binding, we first prepared the complex/HS species in situ by adding 5 equivalents of NaSH to the complex and subsequently added an excess of acetic acid. The best response (in terms of number of cycles possible) was observed for complex **1**: the initial fluorescence intensity, which enhanced upon the addition of HS^−^ and was quenched when acetic acid was added, thus suggesting reversibility of the sensing construct. Figure 3 shows a typical time trace of a solution containing 10 µM of complex **1** when excited at 481 nm.

From the first screening experiments, complex **1** appeared as the most promising in the determination of HS^−^, thus for the next experiments we focused on this complex.

**Figure 4:**
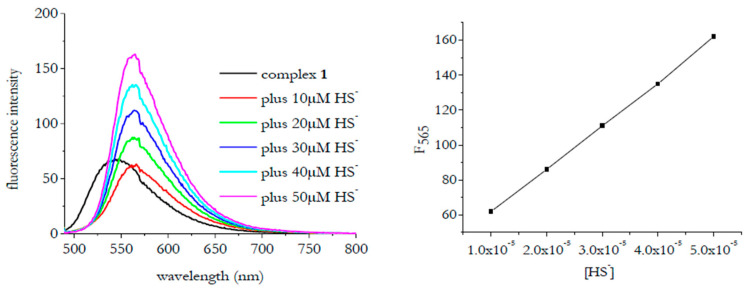
(**Left**) Emission spectra of complex **1** (λ_exc_ = 481 nm) when titrated with NaSH. [Complex **1**] = 1 × 10^−5^ M; end concentration of NaSH varied in the range (1−5) × 10^−5^ M. (**Right**) The fluorescence intensity values of complex **1** at 565 nm have been plotted as a function of the HS^−^ concentration.

To assess whether there was a dependence between the fluorescence intensity of complex **1** and the HS^−^ concentration we performed an experiment where we added increasing amounts of HS^−^ (in the range 10–50 µM) to the complex in DMSO. Figure 4 displays the outcome of this experiment: as visible, a linear correlation was found when plotting the maximum of the fluorescence emission against the HS^−^ concentration, which functioned as calibration of the system.

With the aim of implementing a more stable and above all easily portable device, we immobilized complex **1** on a sol-gel based matrix and checked its fluorescence response to HS^−^ upon immobilization. The fluorescence enhancement observed in solution in the presence of HS^−^ was also retained in the solid state (Figure S10).

Following on from these results, to assess the detection capability of complex **1** in more physiological conditions and to explore the possibility of implementing sol-gel dipping devices for H_2_S visualization, we used ddH_2_O (doubly distilled water) and HBSS buffer where we dissolved HS^−^ at different concentrations, namely 17.8, 178, and 1780 µM, falling into a physiological range [2,54]. Moreover, we tested the sol-gel devices to reveal endogenous HS^−^ produced by the human hepatic HepG2 cell line after 24 h incubation with HBSS media.

**Figure 5:**
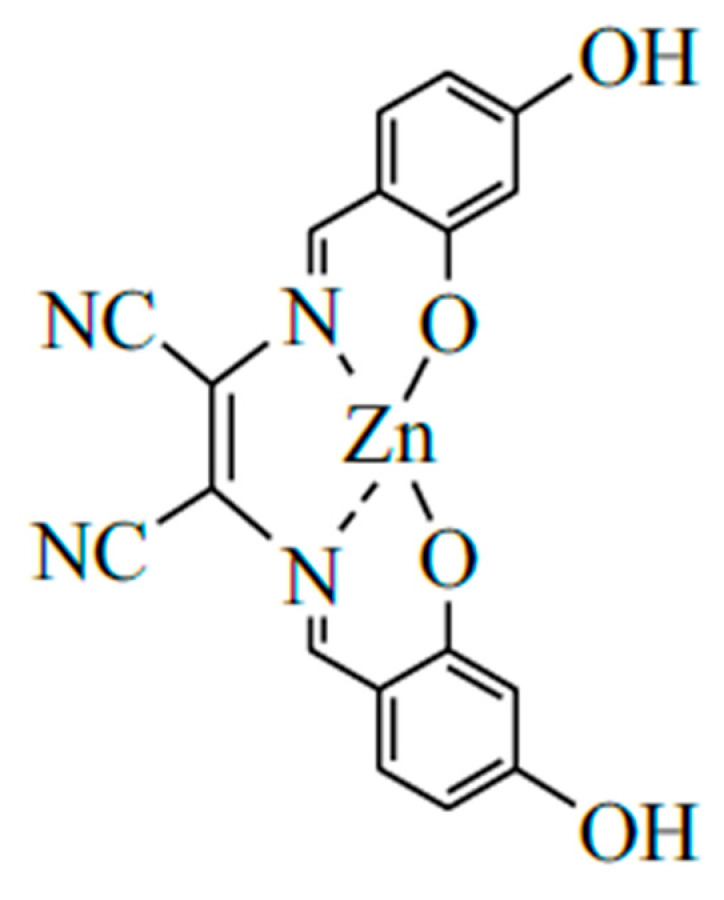
Structure of a model complex.

To optimize the experimental conditions and also for comparative purposes we ran several trial experiments with the complex in Figure 5, which we had reported in the past [41].

**Figure 6:**
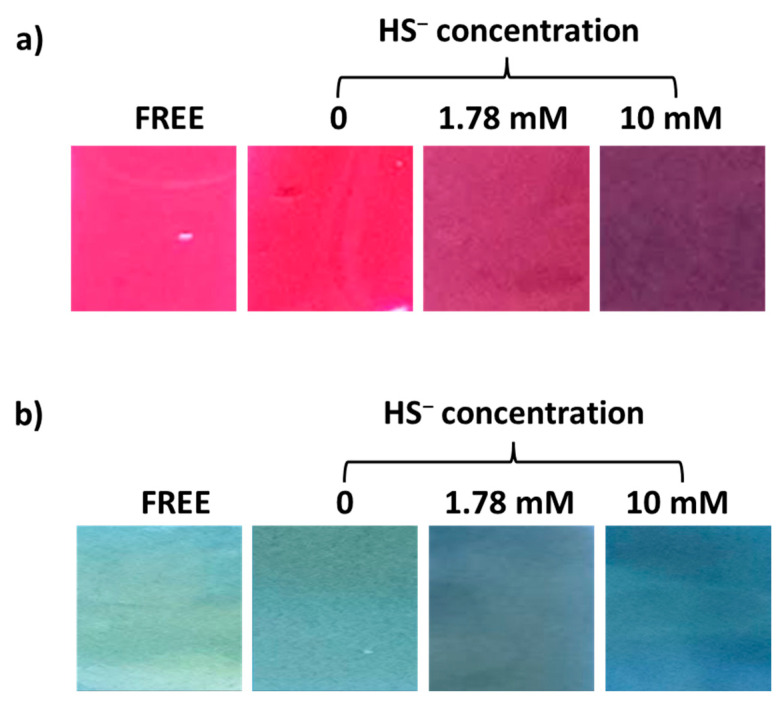
Real color images of a cropped area of the model complex—reported in Figure 5—(**a**) and complex **1** (**b**) loaded in sol-gel matrices after adding increasing amounts of HS^−^ dissolved in H_2_O (0, 1.78, and 10 mM). Non-treated matrices are indicated as FREE.

As observed by experiments reported in Figure 6, preliminary tests indicated that both the complexes loaded in the sol-gel matrices were able to detect different HS^−^ concentrations in ddH_2_O. In fact, an evident color change was visible upon UV irradiation for the model complex (Figure 6a) and complex **1** (Figure 6b).

**Figure 7:**
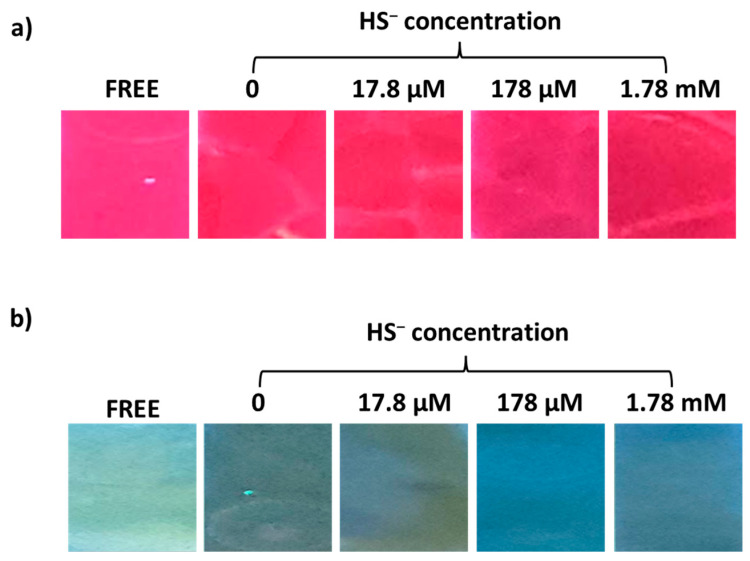
Real color images of a cropped area of the model complex—reported in Figure 5—(**a**) and complex **1** (**b**) loaded in sol-gel matrices after adding increasing amounts of HS^−^ dissolved in HBSS (0, 17.8 µM, 178 µM, and 1.78 mM). Non-treated matrices are indicated as FREE.

Of greater interest, the sol-gel matrices detected lower and physiological HS^−^ concentrations in the HBSS buffer as well, with a chemical composition closer to the biological fluids (Figure 7a,b).

**Figure 8:**
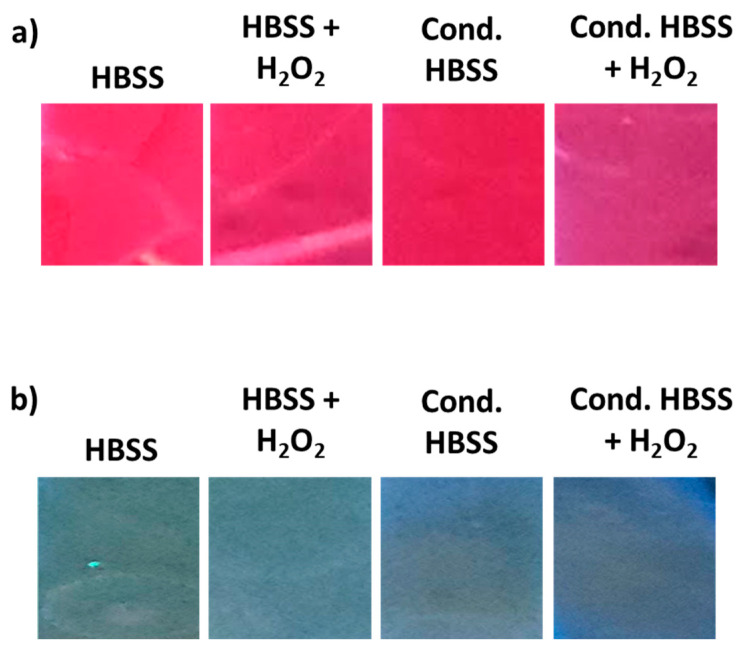
Real color images of a cropped area of the model complex—reported in Figure 5—(**a**) and complex **1** (**b**) loaded in sol-gel matrices after adding cell-conditioned HBSS media. Matrices treated with non-conditioned HBSS (HBSS); non-conditioned medium +800 µM H_2_O_2_ (HBSS+ H_2_O_2_); cell-conditioned HBSS (Cond. HBSS); and cell-conditioned HBSS after 1 h treatment of the HepG2 cells with 800 µM H_2_O_2_ (Cond. HBSS+ H_2_O_2_).

In order to assess the capability of the sol-gel devices to reveal levels of endogenous HS^−^, we used the HBSS media previously incubated with the HepG2 cells for 24 h in the absence and the presence of H_2_O_2_ (as a stimulus to increase HS^−^ production by the cells) [55]. The different chromatic variations of matrices loaded with both complexes were observed (Figure 8a,b). In particular, the matrices in contact with the media conditioned by the cells assumed a color more similar to the samples treated with concentrations of HS^−^ comprised between 178 and 1780 µM (Figure 7). Conversely, the matrices treated with the non-conditioned media showed a less significant color change (Figure 8a,b). Furthermore, one hour treatment of the cells with 800 µM H_2_O_2_ induced a well-evidenced variation of color for both the complexes (Figure 8a,b) which is most likely attributable to an increment in HS^−^ production. As a control, samples treated with non-conditioned medium +800 µM H_2_O_2_ showed a slight color change suggesting no interference by H_2_O_2_ itself. Taken altogether these results demonstrate the great potential of complex **1** and the model complex-loaded sol-gel devices to detect HS^−^ in a biological fluid.

**Investigations into the recognition mechanism of HS^−^ by complexes 1–4**. The proton NMR experiments pointed to the binding of HS^−^ to the zinc centres for all of the complexes under investigation; this was evidenced by the appearance of the high-field SH resonance (visible in all the registered spectra) together with the shift of the signals pattern (see Figures S11–S14) [22,25,39,41,42,43,56]. Similar results, corroborating the binding of HS^−^ to the zinc ions in complexes **1–4**, could be drawn by high-resolution MALDI experiments (Figures S15–S18). In addition, MS spectrometry clearly indicated the formation of 1:1 HS^−^ adducts for complexes **1–4**. The reversibility of HS^−^ binding in the presence of acetic acid with the concomitant modulation of the fluorescence response (see Figure 3) represented a further confirmation that ‘a coordinative mechanism’ of recognition was operative.

**Figure 9:**
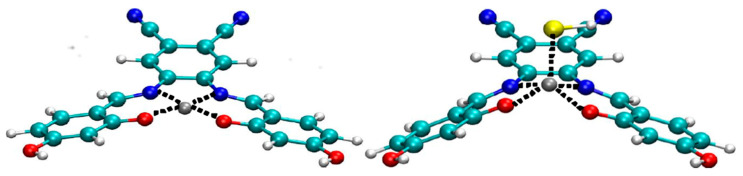
Optimized geometries for complex **1** (**left**) and its adduct with HS^–^ (**right**).

**Computational study**. As we were willing to rationalize the photophysical properties of this new family of complexes, we focused on complexes **1** and **4** and performed a computational analysis at the time dependent density functional theory (TD-DFT) level. The minimum energy geometries of complex **1,** complex **4** and of their HS^–^ adducts (and also considering the possibility of multiple adducts) were computed both for the ground state and for the first excited singlet states. The computed ground state optimum geometries of complex **1** and its HS^–^ complexes are shown in Figure 9 (a similar geometry has been also found for complex **4** and its HS^–^ complex, Figure S19).

Complexes **1** and **4** deviate from the square planar nuclear configuration observed for salen-analogues [41,42], with the metal atom in the plane of the ligand (see Figure 9). The formation of the single adduct is predicted to be exergonic (Δ*E* = −0.86 eV for complex **1**, Δ*E* = −0.92 eV for complex **4**), whereas the double adduct is not predicted to be a stable species, as confirmed by the DFT computation where the second HS^–^ was moved away from the metal centre during the geometry optimization.

The emission from S_1_ was predicted to be electric dipole allowed for both complex **1** and complex **4** and for their HS^−^ adducts. The computed vertical and adiabatic excitation energies are reported in Table 1, together with the oscillator strengths for the S_1_ ← S_0_ transitions.

For complex **1**, three electric dipole transitions were predicted at approximately 307, 349 and 384 nm; in fair agreement with the experimental absorption spectra (see Figure 1). However, a meaningful comparison between the predicted and the observed absorption spectra would require a band-shape simulation, with the computations of Franck-Condon integrals [57] which was far beyond the qualitative purposes of the present computational analysis.

Since emission from S_1_ are electric dipole allowed transitionsfor all the species under study, the different behaviour observed for complex **1** and complex **4** and for their HS^–^ complexes had to be related with the possible existence of non-radiative decay paths. We have thus investigated the energy location of the lowest triplet states, which could be responsible for the different fluorescence quantum yields of complex **1** and complex **4** and their HS^–^ adducts (Table S1).

**Figure 10:**
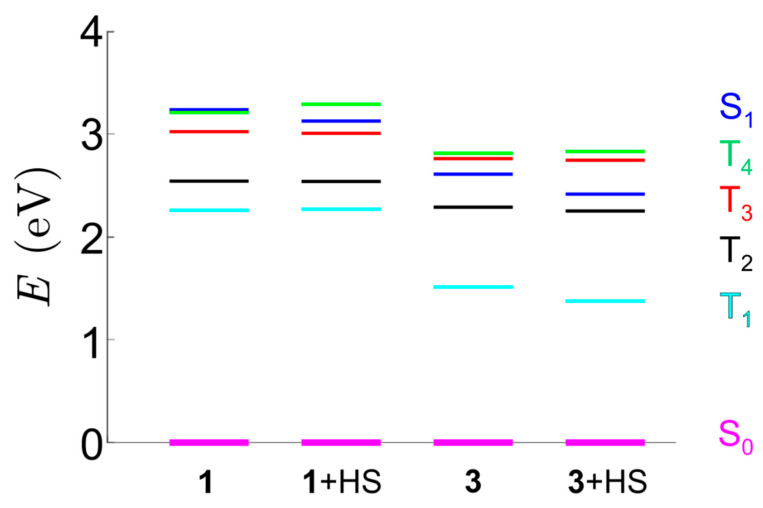
Computed energies (*E*, eV) of the ground state (S_0_) first excited singlet (S_1_), and four lowest energy triplet states, evaluated at the S_0_ geometry for complex **1** and complex **4**, and their HS^–^ adducts.

The energies of the four lowest triplet states are reported in Figure 10; T_5_ lies always above in energy than S_1_ and therefore it should not be involved in non-radiative decay paths. T_1_ is significantly lower in energy than S_1_ for all the species and therefore, based on the energy gap rule, the direct transition S_1_ **→** T_1_ and should not be an efficient decay path. The triplet states closer in energy to S_1_ are T_3_ and T_4_ for complex **1** and T_2_ for complex **4**.

As concerns complex **1**, the S_1_ **→** T_4_ transition was slightly exergonic (10 meV), but it became endergonic (160meV) when the HS^−^ was coordinated (Figure 10). This suggests that quenching of fluorescence via S_1_ **→** T_4_ is possible in the isolated complex, whereas fluorescence is recovered in the presence of HS^−^ in solution, which is in line with the experimental observations (see Figure 2).

On the contrary for complex **4**, the S_1_ **→** T_2_ transition was exoergonic both before and after coordination, however being more favoured after HS^–^ coordination, as demonstrated by the lower energy difference between the electronic states (Figure 10) and by the significantly higher spin-orbit coupling for S_1_ **→** T_2_ (see Table 2). Thus, a strong quenching of fluorescence is to be expected in the presence of HS^–^, as was indeed observed (see Figure 2).

## 4. Conclusions

A new family of salen-based zinc complexes with π-extended structures were prepared, which can be used for direct fluorescence-based and colorimetric detection of HS^−^. The fluorescence modulation observed during the reaction of these complexes with HS^−^ was the result of the binding of the target molecule to the zinc centers as demonstrated by the NMR and the MALDI experiments. This study further demonstrates the value of the coordinative-based approach as a valid strategy for the development of HS^−^ sensors. A computational analysis at the time dependent density functional theory (TD-DFT) level indicated that the different fluorescence responses observed for complex **1** and complex **4** in the presence of HS^–^ had to be related with the possible existence of non-radiative decay paths.

The successful immobilization of the title complexes in the sol-gel matrices paves the way for the development of more stable and easy-portable dipping devices for H_2_S visualization. In addition, our experiments demonstrated the great potential of complex **1** and the model complex-loaded sol-gel devices to detect HS^−^ in a biological fluid.

To our knowledge this is the first study in which the sol gel methodology was used in combination with the metal-based complexes to implement smart devices to be used in the fast growing applications of the H_2_S sensing field.

## Figures and Tables

**Table 1:** Computed vertical and adiabatic excitation energies (eV) and oscillator strength for the S_1_ ← S_0_ transitions.

	Vertical	Adiabatic	Oscillator Strength
**1**	3.22	2.64	0.57
**1** + HS^−^	3.13	2.67	0.42
**4**	2.61	2.21	0.96
**4** + HS^−^	2.42	2.11	0.80

**Table 2:** Spin-Orbit coupling elements (cm^−1^) for complex **1**, complex **4** and their adducts.

	SOC T_2_-S_1_	SOC T_3_-S_1_	SOC T_4_-S_1_
**1**	1.23	10.0	14.1
**1** + HS^−^	45.0	29.2	10.5
**4**	16.59845	5.06933	2.80429
**4** + HS^−^	95.8	240	71.5

## Supplementary Materials

The following are available online at: https://www.mdpi.com/article/10.3390/s23042023/s1, Figure S1: ^1^H NMR spectrum of complex 1 in DMSO-d6, Figure S2: ^1^H NMR spectrum of complex 2 in DMSO-d6, Figure S3: ^1^H NMR spectrum of complex 3 in DMSO-d6, Figure S4: ^1^H NMR spectrum of complex 4 in DMSO-d6, Figure S5: Enlargement of the MALDI of complex 1, Figure S6: Enlargement of the MALDI of complex 2, Figure S7: Enlargement of the MALDI of complex 3, Figure S8: Enlargement of the MALDI of complex 4, Figure S9: Real color images of DMSO solutions of complexes 4, Figure S10: Fluorescence intensity time trace of complex 4 in SOL-GEL matrix, Figure S11: ^1^H NMR spectra of complex 1 in the presence of NaHS, Figure S12: ^1^H NMR spectrum of complex 2 in the presence of NaHS, Figure S13: ^1^H NMR spectrum of complex 3 in the presence of NaHS, Figure S14: ^1^H NMR spectrum of complex 4 in the presence of NaHS, Figure S15: MALDI spectrum of complex 1 in the presence of NaHS, Figure S16: MALDI spectrum of complex 2 in the presence of NaHS, Figure S17: MALDI spectrum of complex 3 in the presence of NaHS, Figure S18: MALDI spectrum of complex 4 in the presence of NaHS, Figure S19: Optimized geometries for complex 1 and its adduct with HS−, Table S1: Photophysical features of complexes 1 and 4.
