# Supplementary material for: Sol-Gel Dipping Devices for H2S Visualization
Source: Sensors (Basel). 2023 Feb 10;23(4):2023. doi: 10.3390/s23042023 (PMC9965526; doi:10.3390/s23042023)
Supplement: Supplementary file 1 [file sensors-23-02023-s001.zip › Figure S10.pdf]

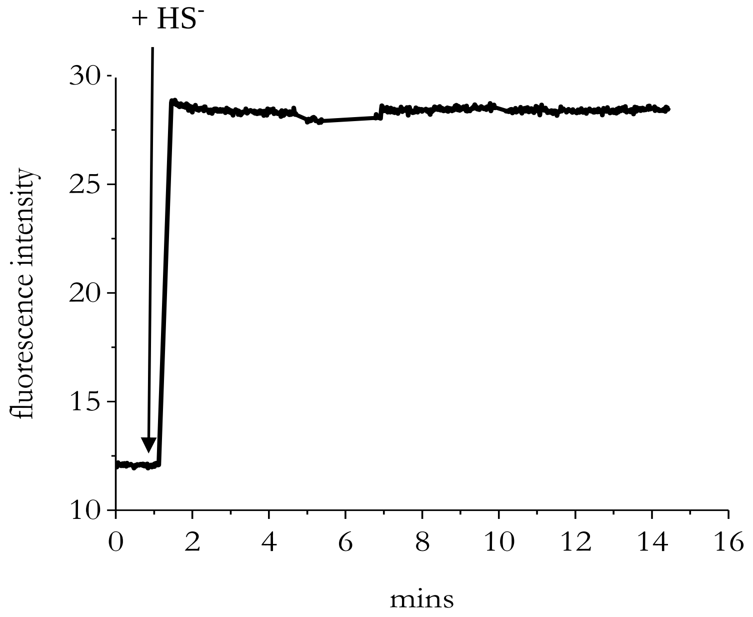

Figure S10. Fluorescence intensity time trace at 550 nm (exc 481 nm) of complex **1** entrapped into a TMOS-based sol–gel immobilized on a quartz support upon addition of HS-. During the measurement the sensing device was placed in water at room temperature. The fluorescence was recorded while HS- was added (to a final concentration of 300 μM) to the solution.
