# Supplementary material for: Sol-Gel Dipping Devices for H2S Visualization
Source: Sensors (Basel). 2023 Feb 10;23(4):2023. doi: 10.3390/s23042023 (PMC9965526; doi:10.3390/s23042023)
Supplement: Supplementary file 1 [file sensors-23-02023-s001.zip › Figure S14.pdf]

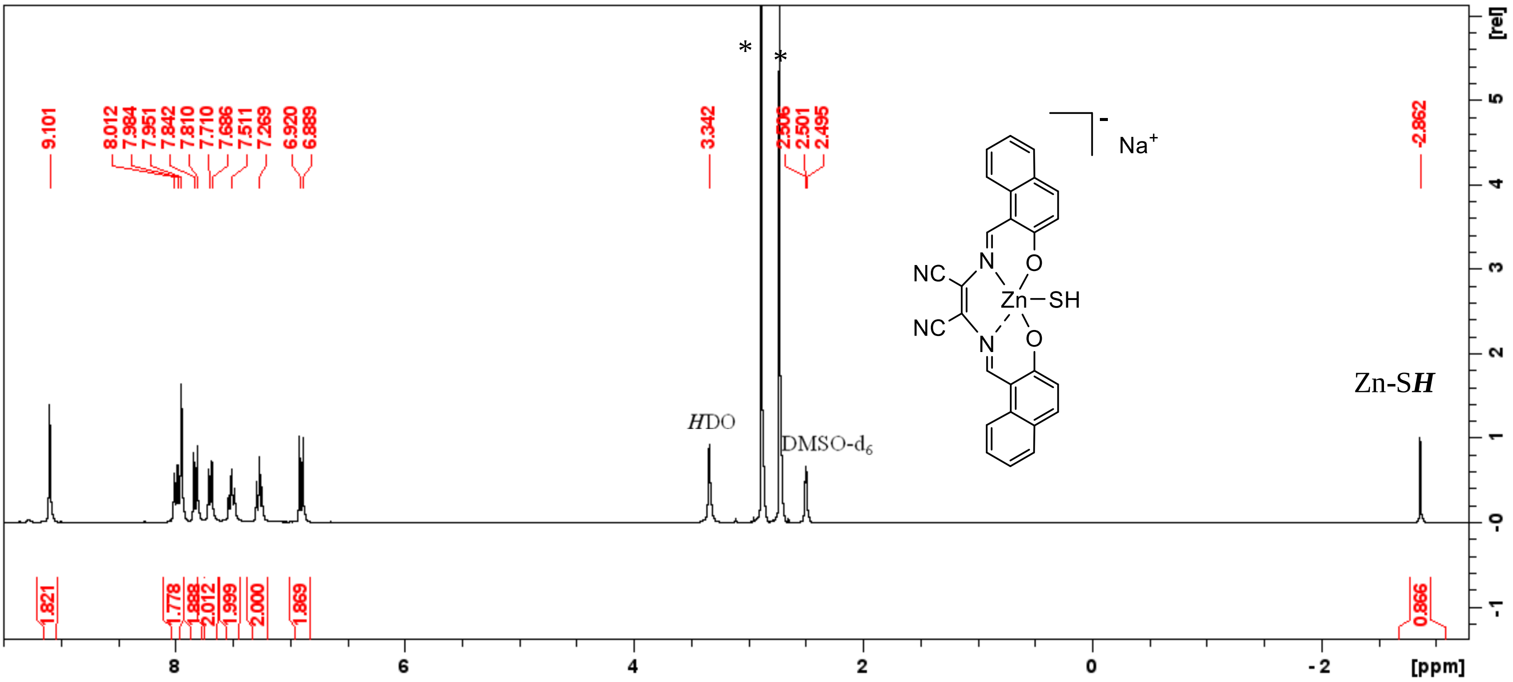

Figure S14. ^{1}H NMR spectrum of complex **4** in DMSO-d_6 after the addition of an excess of HS^-. [complex **4**] = 5×10^{-2} M; [NaSH] = 0.1 M. * = DMF used for the synthesis.
