# Supplementary material for: Sol-Gel Dipping Devices for H2S Visualization
Source: Sensors (Basel). 2023 Feb 10;23(4):2023. doi: 10.3390/s23042023 (PMC9965526; doi:10.3390/s23042023)
Supplement: Supplementary file 1 [file sensors-23-02023-s001.zip › Figure S18.pdf]

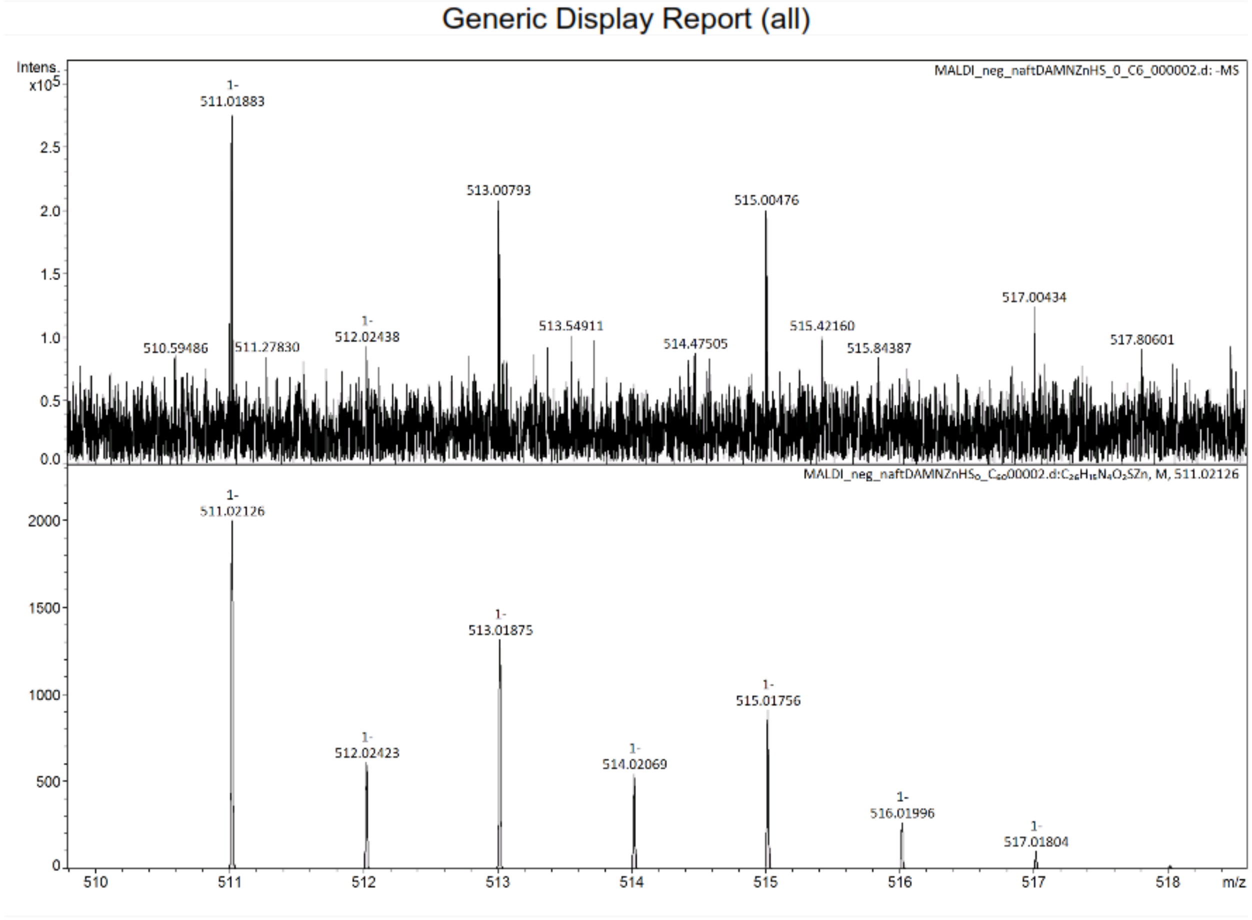

Figure S18. Enlargement of the MALDI spectrum of complex **4** in the presence of NaHS in CH_3CN with 1% DMSO. The upper trace is the experimental trace whereas the lower is the theoretical one.
