# Supplementary material for: Sol-Gel Dipping Devices for H2S Visualization
Source: Sensors (Basel). 2023 Feb 10;23(4):2023. doi: 10.3390/s23042023 (PMC9965526; doi:10.3390/s23042023)
Supplement: Supplementary file 1 [file sensors-23-02023-s001.zip › Figure S3.pdf]

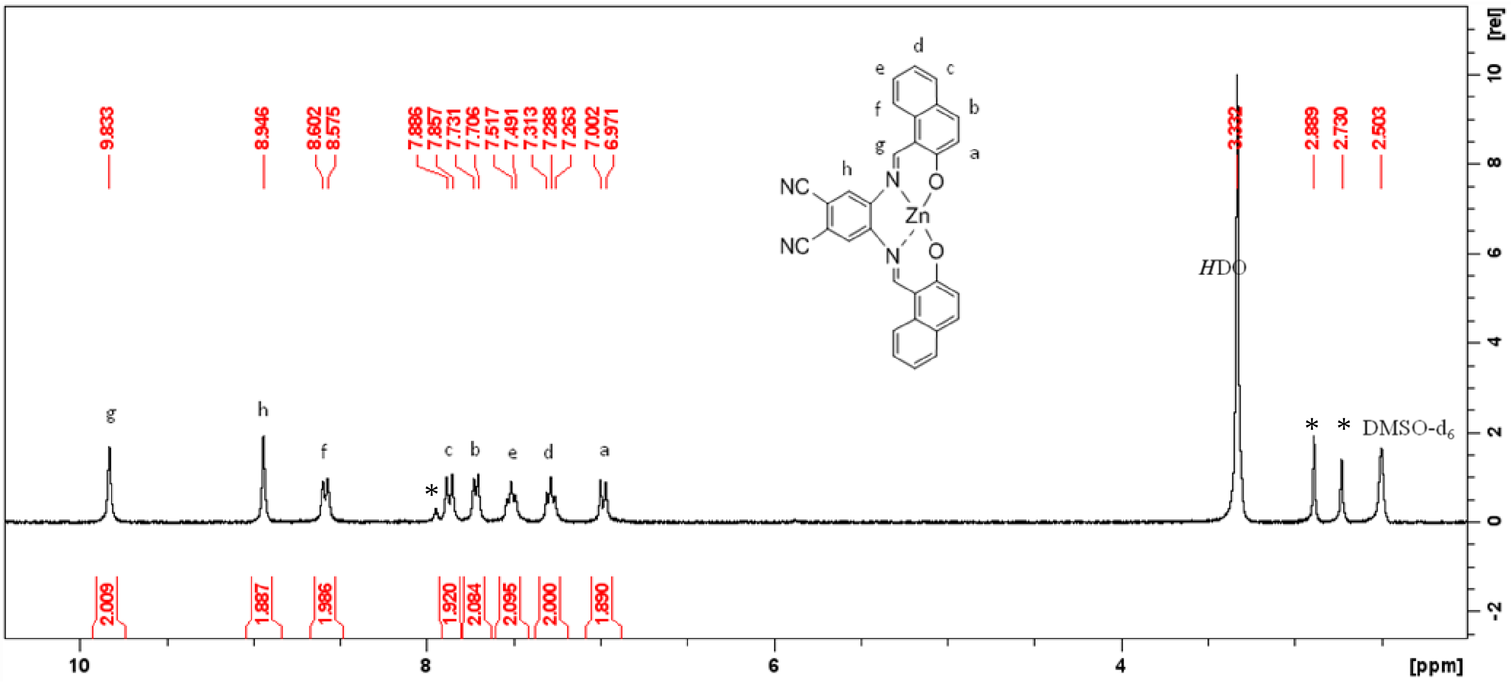

Figure S3. ^{1}H NMR spectrum of complex **3** in DMSO-d$_6$. [complex **3**] = 50×10^{-3} M. * = DMF used for the synthesis.
