# Supplementary material for: Sol-Gel Dipping Devices for H2S Visualization
Source: Sensors (Basel). 2023 Feb 10;23(4):2023. doi: 10.3390/s23042023 (PMC9965526; doi:10.3390/s23042023)
Supplement: Supplementary file 1 [file sensors-23-02023-s001.zip › Figure S4.pdf]

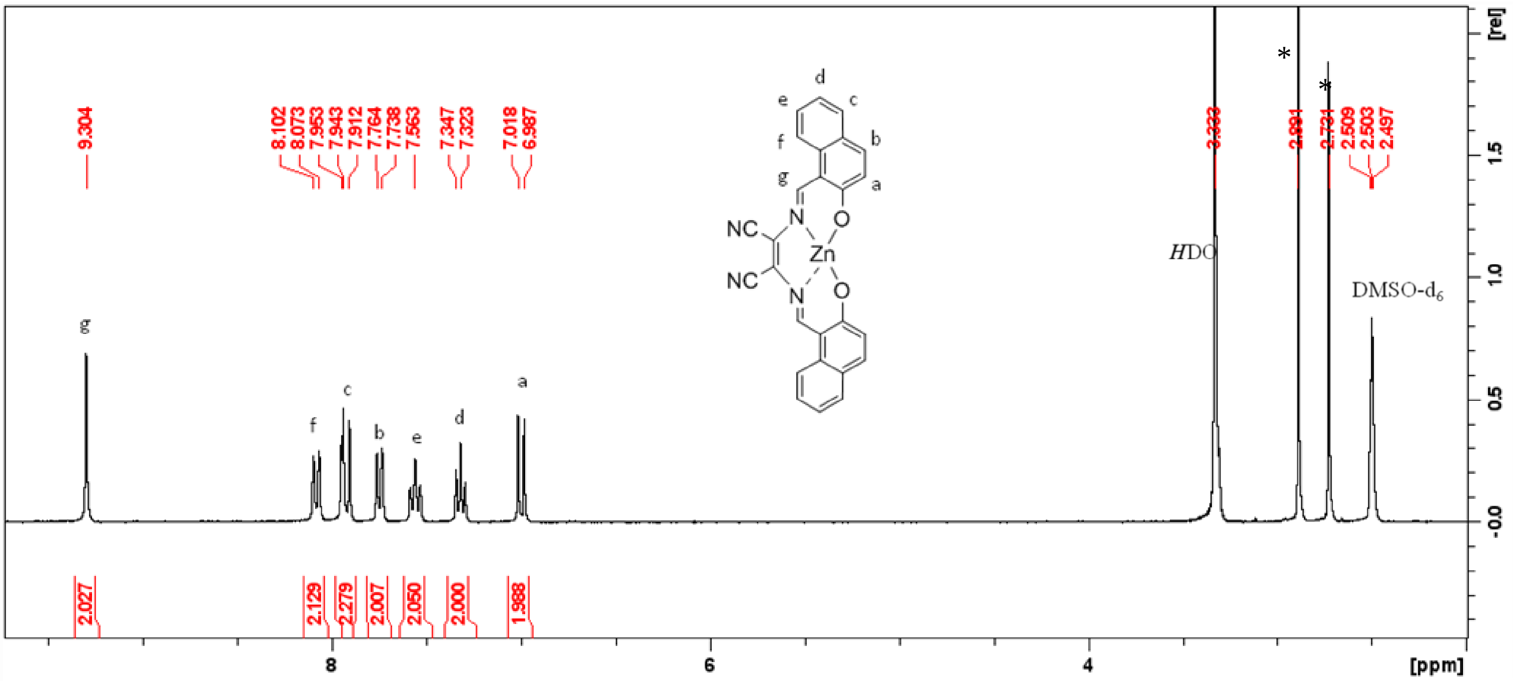

Figure S4. ^{1}H NMR spectrum of complex **4** in DMSO-d_6. [complex **4**] = 50×10^{-3} M. * = DMF used for the synthesis.
