# Supplementary figures and images for: Sol-Gel Dipping Devices for H2S Visualization
Source: Sensors (Basel). 2023 Feb 10;23(4):2023. doi: 10.3390/s23042023 (PMC9965526; doi:10.3390/s23042023)

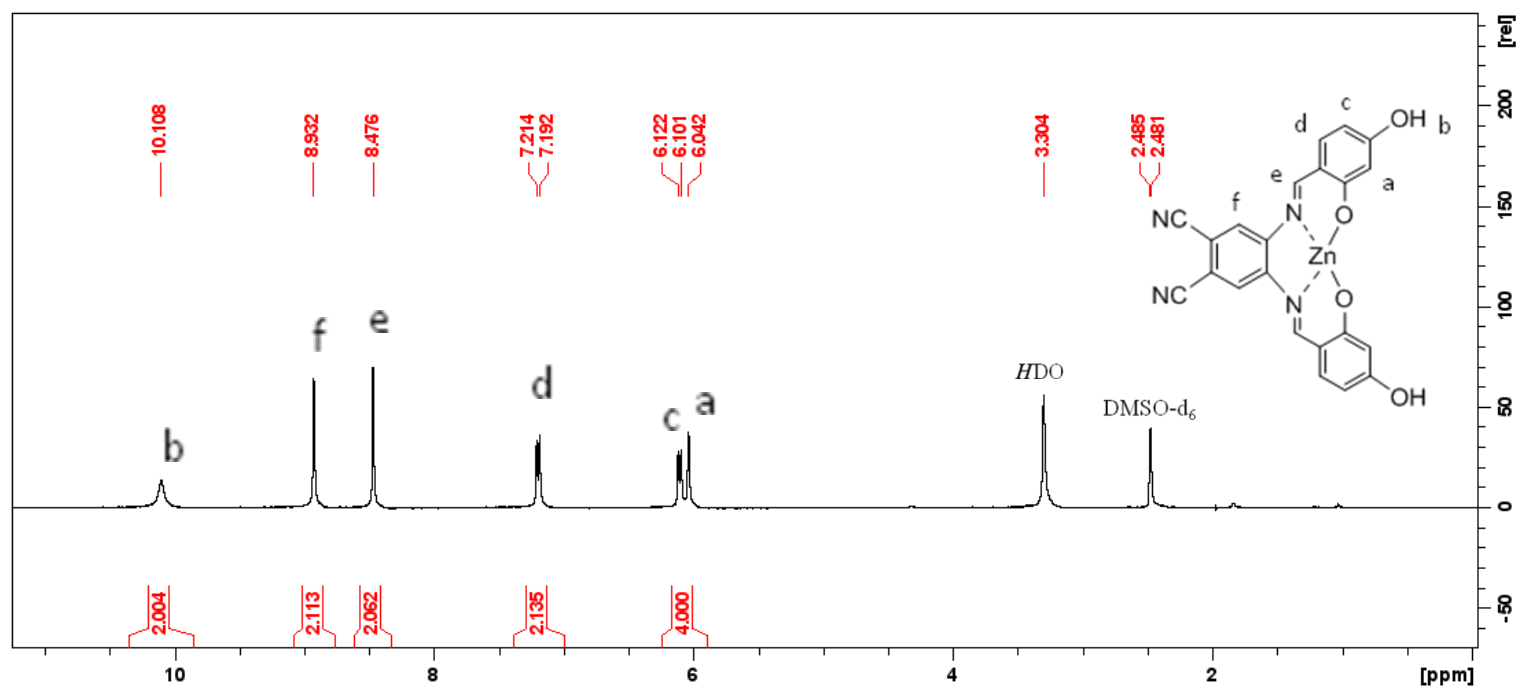

**Figure S1.**  $^1\text{H}$  NMR spectrum of complex **1** in  $\text{DMSO-d}_6$ .  $[\text{complex } \mathbf{1}] = 50 \times 10^{-3} \text{ M}$ .

Supplement: Supplementary file 1 [file sensors-23-02023-s001.zip › Figure S1.pdf]

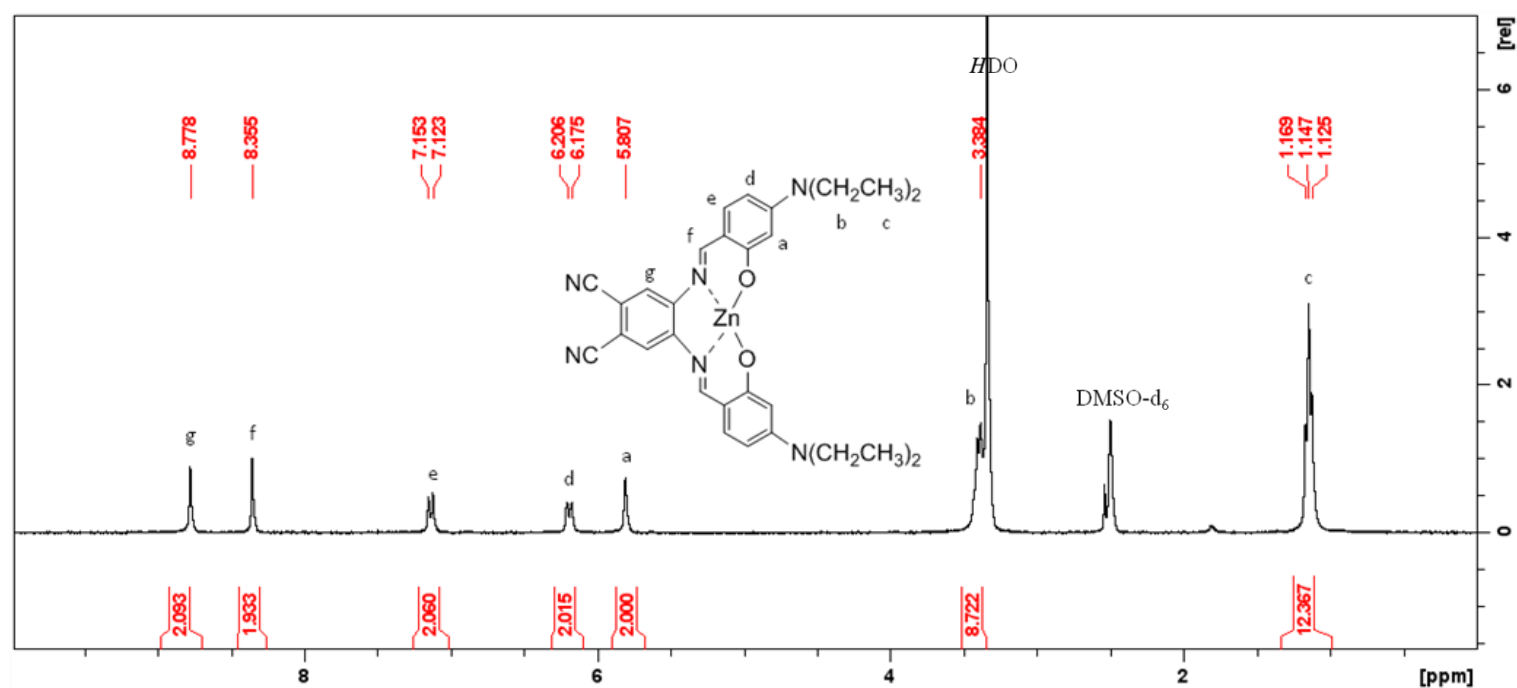

**Figure S2.**  $^1\text{H}$  NMR spectrum of complex **2** in  $\text{DMSO-d}_6$ .  $[\text{complex } \mathbf{2}] = 50 \times 10^{-3} \text{ M}$ .

Supplement: Supplementary file 1 [file sensors-23-02023-s001.zip › Figure S2.pdf]
